# Establishment of international autoantibody reference standards for the detection of autoantibodies directed against PML bodies, GW bodies, and NuMA protein

**DOI:** 10.1515/cclm-2020-0981

**Published:** 2020-08-10

**Authors:** Bing Zheng, Rodrigo A. Mora, Marvin J. Fritzler, Minoru Satoh, Donald B. Bloch, Ignacio Garcia-De La Torre, Katherine Boylan, Kathryn Kohl, Mark H. Wener, Luis E. C. Andrade, Edward K. L. Chan

**Affiliations:** Department of Oral Biology, University of Florida, Gainesville, FL, USA; Department of Laboratory Medicine, Renji Hospital, School of Medicine, Shanghai Jiao Tong University, Shanghai, PR China; Department of Oral Biology, University of Florida, Gainesville, FL, USA; Department of Medicine, Cumming School of Medicine, University of Calgary, Calgary, Canada; Department of Clinical Nursing, University of Occupational and Environmental Health, Kitakyushu, Japan; Division of Rheumatology, Allergy and Immunology, Department of Medicine, Massachusetts General Hospital and Harvard Medical School, Boston, MA, USA; Department of Immunology and Rheumatology, Hospital General de Occidente and University of Guadalajara, Guadalajara, Mexico; Scientific & Clinical Affairs, Plasma Services Group Inc., Huntingdon Valley, PA, USA; Scientific & Clinical Affairs, Plasma Services Group Inc., Huntingdon Valley, PA, USA; Division of Rheumatology and Department of Laboratory Medicine, University of Washington, Seattle, WA, USA; Rheumatology Division, Escola Paulista de Medicina, Universidade Federal de São Paulo, São Paulo, Brazil; Immunology Division, Fleury Laboratories, São Paulo, Brazil; Department of Oral Biology, University of Florida, 1395 Center Drive, Gainesville, FL, 32610-0424, USA.

**Keywords:** autoimmunity, GW body, multiple nuclear dots, NuMA, reference materials

## Abstract

**Objectives:**

Reference materials are important in the standardization of autoantibody testing and only a few are freely available for many known autoantibodies. Our goal was to develop three reference materials for antibodies to PML bodies/multiple nuclear dots (MND), antibodies to GW bodies (GWB), and antibodies to the nuclear mitotic apparatus (NuMA).

**Methods:**

Reference materials for identifying autoantibodies to MND (MND-REF), GWB (GWB-REF), and NuMA (NuMA-REF) were obtained from three donors and validated independently by seven laboratories. The sera were characterized using indirect immunofluorescence assay (IFA) on HEp-2 cell substrates including two-color immunofluorescence using antigen-specific markers, western blot (WB), immunoprecipitation (IP), line immunoassay (LIA), addressable laser bead immunoassay (ALBIA), enzyme-linked immunosorbent assay (ELISA), and immunoprecipitation–mass spectrometry (IP-MS).

**Results:**

MND-REF stained 6–20 discrete nuclear dots that colocalized with PML bodies. Antibodies to Sp100 and PML were detected by LIA and antibodies to Sp100 were also detected by ELISA. GWB-REF stained discrete cytoplasmic dots in interphase cells, which were confirmed to be GWB using two-color immunofluorescence. Anti-Ge-1 antibodies were identified in GWB-REF by ALBIA, IP, and IP-MS. All reference materials produced patterns at dilutions of 1:160 or greater. NuMA-REF produced fine speckled nuclear staining in interphase cells and staining of spindle fibers and spindle poles. The presence of antibodies to NuMA was verified by IP, WB, ALBIA, and IP-MS.

**Conclusions:**

MND-REF, GWB-REF, and NuMA-REF are suitable reference materials for the corresponding antinuclear antibodies staining patterns and will be accessible to qualified laboratories.

## Introduction

Autoantibody assays are often used to assist in the evaluation of patients suspected of having a wide spectrum of autoimmune disorders. In clinical laboratories, the indirect immunofluorescence assay (IFA) using the HEp-2 cell substrate (HEp-2 IFA) was regarded the “gold standard” test for antinuclear antibody (ANA) screening by the American College of Rheumatology [1]. To promote standardization of HEp-2 IFA reporting, thirty anti-cell staining patterns (AC-0 to AC-29) have been described by the International Consensus on ANA Patterns (ICAP) (www.anapatterns.org) and their clinical relevance summarized to benefit clinicians in their daily work [2]. However, the identification of some esoteric patterns remains challenging for many laboratories [3]. Many factors may affect HEp-2 IFA testing including variations in different commercial HEp-2 kits, sensitivity of microscope settings, and pattern reading experience of technical staff.

The development and validation of robust, certified, and traceable reference standards is a critical element in clinical laboratory quality assurance analytics. There are already 20 ANA reference materials available from the Autoantibody Standardization Committee for various ANA patterns [4]. Typically, sufficient quantity of plas-mapheresis sample obtained from one single donor showing specific ANA patterns and/or antigen specificity is selected for further analysis and then validated on different platforms in multiple expert autoantibody testing laboratories worldwide. These reference materials established by the Autoantibody Standardization Committee are now distributed free of charge by Plasma Services Group (PSG, Huntingdon Valley, PA, USA; https://www.plasmaservicesgroup.com/). Notably, there is still an urgent need to address other less commonly seen ANA patterns, which are crucial in training, documenting proficiency, and standardizing the interpretation of HEp-2 IFA for optimal clinical testing as well as research studies.

The multiple nuclear dots (MND) IFA pattern AC-6, is characterized by 6-20 discrete dots in interphase nuclei. The major target antigens of anti-MND are promyelocytic leukemia protein (PML) bodies including the protein PML, the “speckled 100kD” protein (Sp100) [5], and the PML bodies-associated nuclear matrix protein NXP-2 [6, 7]. Antibodies directed against PML and Sp100 are associated with primary biliary cholangitis (PBC) [8-10] and the presence of these antibodies assists in the diagnosis of patients who are anti-mitochondrial antibody (AMA)-negative [11]. The Sp100 proteins are represented by at least four splice variants: Sp100A, Sp100B, Sp100C, and Sp100-HMG. All of the variants contain the immunoreactive domain and show aberrant electrophoretic mobility as a 100 kDa protein [12]. Reports have shown that anti-Sp100 has low sensitivity of 20-40% [8, 13, 14], but a remarkably high specificity (>95%) for PBC [14, 15]. Anti-PML antibodies have a relatively lower prevalence compared to anti-Sp100 in PBC patients and the majority of anti-PML seropositive sera have simultaneous reactivity to Sp100 [9, 16, 17]. The presence of anti-Sp100 autoantibody and cooccurrence of anti-Sp100 and in some reports anti-PML autoantibodies have been reported to correlate with unfavorable disease outcomes [9, 17, 18]. Another autoantigen NXP2, also known as microrchidia family CW-type zinefinger 3 (MORC3), is also enriched in PML bodies [19]. The consensus on the clinical relevance for AC-6 pattern has been summarized recently by ICAP [2].

The cytoplasmic discrete dots pattern AC-18 primarily represents staining of GW bodies (GWB) in the cytoplasm of interphase cells. GWB are distinct from endosomes, lysosomes, peroxisomes, and the Golgi complex [20, 21]. These foci are rich in GW182, a 182 kDa glycine-tryptophan (GW) repeats-rich autoantigen that was initially identified using the serum of a patient with sensory ataxic polyneuropathy [21]. Subsequent studies showed that GWB are the same structures as the cytoplasmic processing bodies, which are involved in mRNA processing and degradation [22, 23]. Several known autoantigens, including GW182 [20], Argonaute2 (Ago2) [24], Ge-1/HEDLS/EDC4, and RAP55/LSm14 [25] are localized to GWB. GW182 is characterized by 39 repeats of glycine (G)-tryptophan (W) dipeptide motifs and multiple Ago2-binding domains, which are involved in miRNA-mediated gene silencing. Ge-1, a ~160 kDa protein also known as HEDLS and EDC4, was initially identified as an autoantigen in a patient with Sjögren’s syndrome (SjS) in 1994 [26]. Ge-1 is an enhancer of the decapping protein complex, which includes Dcp1 and Dcp2, Rck/p54, and hEDC3 proteins [27]. The clinical diagnoses associated with the presence of anti-GWB antibodies were reported with SjS and mixed motor/sensory neuropathy, but these antibodies have also been detected in patients with systemic lupus erythematosus (SLE), PBC, and other clinical conditions [21, 28, 29]. The summary of ICAP on clinical relevance for AC-18 has been published [2].

Antibodies that produce the nuclear mitotic apparatus antigen (NuMA)-like pattern (AC-26) stain both the nucleoplasm in interphase cells and mitotic spindle in dividing cells [6]. Anti-NuMA antibodies recognize centrophilin associated with the mitotic spindle apparatus [30]. NuMA/centrophilin is an abundant 236 kDa protein, which plays a critical role in mitotic spindle formation, chromosome separation, and nuclear reassembly [31]. Although anti-NuMA has an estimated prevalence of <1% among sera screened for HEp-2 IFA [31-34], it has strong clinical associations with systemic autoimmune diseases. Anti-NuMA autoantibodies are more commonly associated with SjS and SLE, but may also be present in patients with rheumatoid arthritis (RA), undifferentiated connective tissue disease, mixed connective tissue disease, and in non-autoimmune conditions such as infections or malignancies [31, 34]. Although AC-6, AC-18, and AC-26 patterns are among the lower prevalent AC patterns in populations studied and ICAP has classified these patterns at the “expert reporting level” – more challenging and reportable only when observers or technologists have attained the experience – there is a critical need to have reliable and accessible reference materials to facilitate identification of these patterns.

In this report, we describe the characterization and development of reference materials for identifying autoantibodies to MND, GWB, and NuMA respectively, which we refer to as the MND reference material (MND-REF), GWB reference material (GWB-REF), and NuMA reference material (NuMA-REF). These reference materials have been tested by seven independent autoantibody-expert laboratories, using HEp-2 IFA, immunoprecipitation (IP), western blot (WB), enzyme-linked immunosorbent assay (ELISA), chemiluminescence immunoassay (CLIA), line immunoassay (LIA), and addressable laser bead immunoassay (ALBIA).

## Materials and methods

### Patient information and reference sample preparation

MND-REF, GWB-REF, and NuMA-REF are undiluted, defibrinated plasma collected and processed by PSG from single donors. MND-REF donor was a 53-year-old female with PBC – autoimmune hepatitis overlap syndrome. The GWB-REF donor was a 46-year-old female diagnosed with systemic sclerosis. NuMA-REF donor was a 68-year-old female diagnosed with RA. After evaluating the suitability of the samples as reference materials by participating laboratories, the samples were lyophilized and distributed to participated laboratories to determine whether the lyophilization had any effect on autoantibody reactivities.

### Ethical approval

Research using only de-identified human samples in this study complies with all relevant national regulations and institutional policies in compliance with the Helsinki Declaration of 1975 as revised in 2013. Informed consent was obtained by PSG in the collection of reference materials and/or was approved by appropriate institutional review boards.

### HEp-2 indirect immunofluorescence assay

All three reference materials were analyzed using IFA on HEp-2 cell substrate slides from Inova Diagnostics (San Diego, CA, USA), Bio-Rad (Hercules, CA, USA), Zeus Scientific (Raritan, NJ, USA), Bion (Des Plaines, IL, USA), MBL (Nagoya, Japan), as previously described [35, 36]. All three reference materials were tested at a dilution of 1:40 and two-fold serial dilutions up to 1:1,280. Secondary antibodies employed are listed in Supplementary Table S1.

Double staining using MND-REF and GWB-REF was performed on HEp-2 cell slides (Inova) to observe costaining of subcellular structures. MND-REF (1:100) costaining was tested using rabbit anti-PML (1:300, provided by Dr. Alexander Ishov, Department Anatomy and Cell Biology, University of Florida). GWB-REF (1:100) costaining was tested with rabbit anti-Rck/p54 antibodies (1:100, MBL International, Woburn, MA). Alexa Fluor 568-conjugated donkey anti-rabbit IgG (H + L) (1:500; A10042, Thermo Fisher) and Alexa Fluor 488-conjugated goat anti-human IgG (H + L) (1:500; A11013, Thermo Fisher) were used as secondary antibodies. All the slides were mounted using Vectashield Mounting Medium with 4′,6-diamidino-2-phenylindole (VECTOR Laboratories, Burlingame, CA). Fluorescent images were manually imaged with a 40× objective on an Olympus BX53 fluorescence microscope.

### Immunoprecipitation

Antigens recognized by reference materials and positive control sera were analyzed by IP using extracts from K562 (human erythroleukemia) cells metabolically radiolabeled with ^35^S-methionine/cysteine, as previously described [37,38]. Briefly, cells were labeled for 14 h with 4.2 mCi in 45 mL ^35^S-l-methionine and ^35^S-l-cysteine (NEG772, PerkinElmer, Waltham, MA, USA) and lysed in 0.5 M NaCl NET/IGEPAL CA-630 buffer (500mM NaCl, 2 mM EDTA, 50 mM Tris-HCl, pH 7.5, 0.3% IGEPAL CA-630) containing 0.5 mM PMSF and 0.3 TIU/mL aprotinin. Cell extracts were cleared by centrifugation and immunoprecipitated on Protein A Sepharose beads (17-0780-01, GE Healthcare, Marlborough, MA, USA) coated with IgG from 8 μL of human reference materials. Beads were then washed with 0.5 M NaCl NET/IGEPAL CA-630 buffer. Immunoprecipitated proteins were subjected to SDS-PAGE followed by autoradiographic imaging.

### Western blot

Cultured human acute lymphoblastic leukemia MOLT-4 and HeLa cells were lysed with 2mL Buffer A (0.5 M NaCl, 10 mM Tris-HCl, 1.5 mM MgCl_2_, 0.5% NP-40, pH 7.5) per 10^9^ cells on ice for 15 min and then centrifuged 10 min at 4 °C. Cell lysates were aliquoted and stored in −80 °C until use. For WB, 10 μL of cell lysate was loaded on a 4–12% gradient SDS-PAGE gel and proteins were transferred to nitrocellulose membranes [39]. After blocking with 5% non-fat dry milk for 1 h, strips of the membranes were incubated with reference materials (1:2,000) for 1 h followed by washing step and 1 h incubation with goat F(ab′)_2_ anti-human IgG conjugated to horseradish peroxidase (1:10,000; cat. 2042-05, Southern Biotech, Birmingham, AL, USA) at room temperature. Reactivity was detected using SuperSignal West Pico PLUS chemiluminescent substrate (Cat. 34577, Thermo Fisher).

### Immunoprecipitation – mass spectrometry

Immunoglobulins in 10 μL of each reference material were bound to Dynabeads Protein A (90 μL; Dynal Biotech Inc., Lake Success, N.Y.) at room temperature for 10 min. After washing and then crosslinking with dimethyl pimelimidate dihydrochloride (Sigma), the IgG-bound beads were incubated with MOLT4 whole cell lysate as described above at 4 °C for 1 h. An aliquot of the IP products was first analyzed by SDS-PAGE and silver staining (Pierce™ Silver Stain for Mass Spectrometry, Thermo Fisher). The remaining IP products were analyzed by Nano-liquid chromatography tandem mass spectrometry (Nano-LC/MS/MS) at the University of Florida Mass Spectrometry Research and Education Center.

### Line immunoassay

Aliquots of the three reference materials were screened for 18 autoantibodies (nRNP/Sm, Sm, RNP-70, RNP-A, RNP-C, SSA/Ro60, Ro52, SSB, Scl-70, PM/Scl, Jo-1, CENP-B, PCNA, dsDNA, nucleosomes, histones, ribosomal P-proteins, AMA-M2) using Euroline ANA Profile 5 (Euroimmun, Lübeck, Germany). In addition, MND-REF and GWB-REF were also tested for 13 autoantibodies (RNAPIII subunits RP155 and RP11, fibrillarin, Th/To, Nor90, PDGFR, CENP-B, CENP-A, Ro52, Ku, PM/Scl75, PM/Scl100 and native Scl-70 using SSc-LIA) (Euroimmun), nine autoantibodies (AMA-M2, M2-3E (BPO), Sp100, PML, gp210, LKM-1, LC-1, SLA/LP and Ro-52) using Autoimmune Liver Diseases Profile – LIA (Euroimmun), and 16 autoantibodies (Mi-2α, Mi-2β, TIF1γ, MDA-5, NXP2, SAE1, Ku, PM/Scl100, PM/Scl75, Jo-1, SRP, PL-7, PL-12, EJ, OJ, and Ro52) using Autoimmune Inflammatory Myopathies – LIA (Euroimmun).

### Addressable laser bead immunoassay

The reactivity of the three reference materials with p80-coilin, SMN1, Gemin3, RUVBL1, RUVBL2, GW182, Ge-1, Ago2, and early endosome antigen 1(EEA1), HsEg5, γ-Tubulin, PCNT, CENPF1, CENPF4, NuMA, Enolase1, PLK4, Cep110, Ninein, AMA-Mit3, LKM, SLA, LC1, HK, KL, Sp100, gp210, VCP were tested by ALBIA on a Luminex 100 flow fluorometer (Luminex Corp., Austin, TX, USA) as previously described [21] in the Mitogen Advanced Diagnostics Laboratory (Calgary, AB, Canada). The cutoff for ALBIA was set at three standard deviations above the mean of control samples (median fluorescence intensity, MFI).

### Enzyme-linked immunosorbent assay

Anti-Sp100 ELISA kits and anti-Scl-70 ELISA kits were purchased from Inova and Euroimmun, respectively and performed according to the manufacturer’s instructions.

## Results

The validation of MND-REF, GWB-REF, and NuMA-REF, as appropriate reference materials was performed by seven laboratories affiliated with the Autoantibody Standardization Committee (www.AutoAb.org), a subcommittee of the International Union of Immunological Societies [4]. The results shown here are representative of the data acquired and reported by these laboratories.

### MND-REF validation

**Figure 1:**
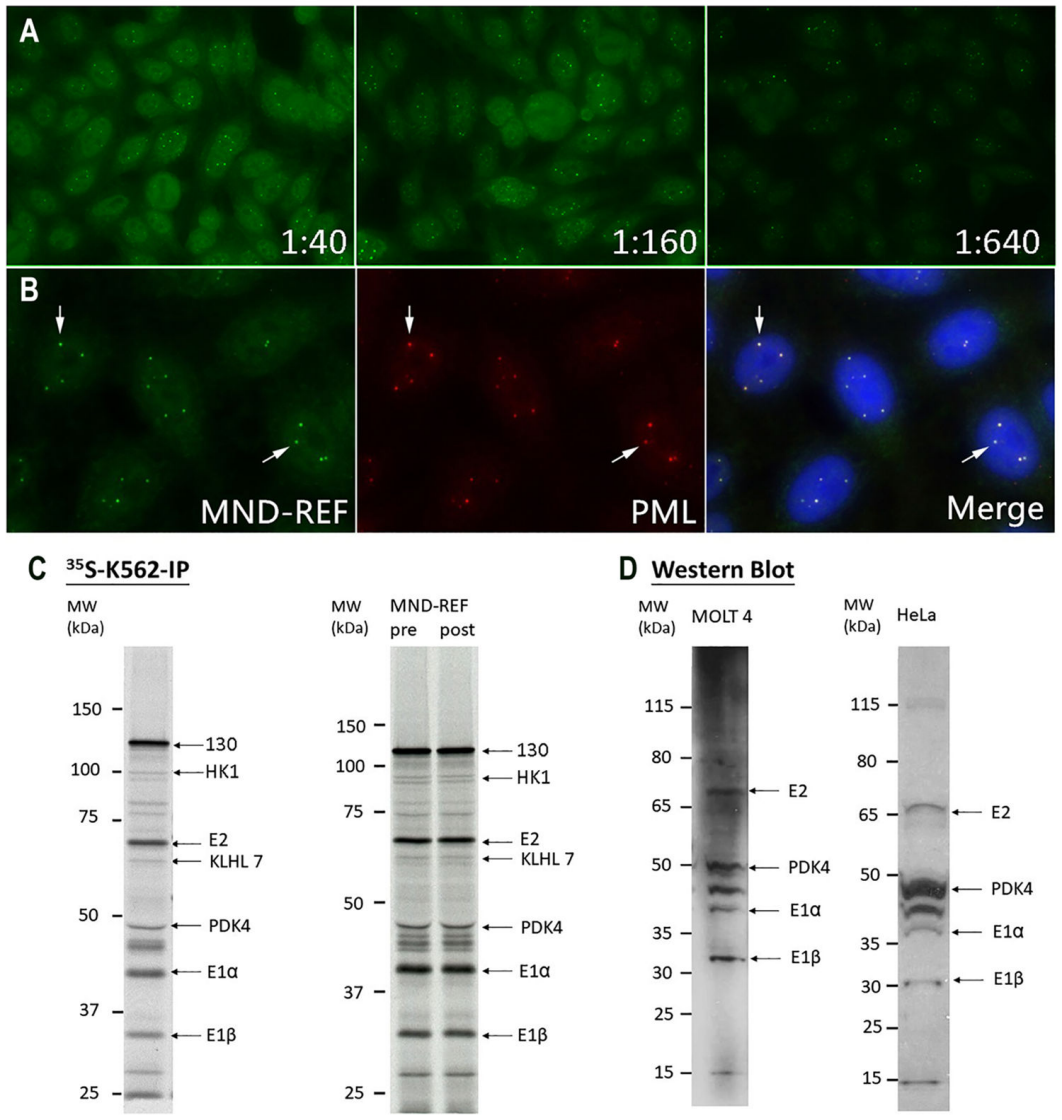
MND-REF validation by HEp-2 IFA, double staining, ^35^S-K562-IP and western blotting. (A) MND-REF was serially diluted from 1:40 to 1:1280 for HEp-2 IFA. Representative images are shown for 1:40, 1:160, and 1:640 dilutions and they all presented 6–20 nuclear discrete dots corresponding to the ICAP AC-6 pattern (https://www.anapatterns.org/view_pattern.php?pattern=6). (B) Double staining of nuclear foci (arrows) identified by MND–REF (green) and rabbit anti-PML (red). (C) Left panel: IP products using ^35^S-methionine-labeled K562 cell extract (^35^S-K562-IP) of MND-REF were analyzed by 8% SDS-PAGE and autoradiography. Right panel: IP of MND-REF pre- and post-lyophilization. The proteins immunoprecipitated by MND-REF corresponded to PIC1/SUMO-1-modified PML, HK1, KLHL 7, PDK4, and protein subunit E2, E1α, and E1β of the pyruvate dehydrogenase complex (PDC) corresponding to proteins identified by IP-MS. (D) Western blotting of MND-REF using MOLT4 (left panel; 1:2,000) or HeLa (right panel; 1:5,000) whole cell lysates. Both showed reactivity to PDK4 and protein subunit E2, E1α, and E1β of PDC corresponding to proteins identified by IP-MS.

MND-REF was first validated by HEp-2 IFA (Figure 1A). The presence of 6–20 discrete nuclear dots per interphase cell, corresponding to the ICAP AC-6 pattern, was reported by all participating laboratories (Supplementary Table S1). Serial dilution of the sample showed a titer of ≥1:320 for AC-6. In addition to AC-6, other patterns were reported including cytoplasmic reticular/AMA pattern (AC-21) in three laboratories using Bion, Biorad and Zeus slides and nuclear fine speckled pattern (AC-4) in three laboratories using Bion, Inova, MBL and in-house HEp-2 slides (Supplementary Table S1). As expected, there are some variations in the images collected from different laboratories. Supplementary Figure S1 shows images illustrating difference in staining from different laboratories as reported in Supplementary Table S1. Note that the differences are not necessarily due only to different HEp-2 substrates used but also affected by secondary antibodies as well as photography settings including exposure and field selection.

Two-color immunofluorescence staining of HEp-2 cells with MND-REF and rabbit anti-PML antiserum confirmed that the dot-like nuclear domains recognized by antibodies in MND-REF co-localized with PML (Figure 1B). In addition, transfection of plasmids encoding green fluorescent protein (GFP)-Sp100 or GFP-PML into HEp-2 cells and staining with mouse anti-GFP antibodies and MND-REF, confirmed that the serum contains antibodies directed against the PML nuclear body (DBB, data not shown).

LIA, ALBIA, and ELISA were also performed to validate the MND-REF. Antibodies directed against both Sp100 and PML were detected in the serum by LIA. Reactivity to Sp100 was also confirmed by ELISA and ALBIA with values of 170 RU and 1083 MFI, respectively (cutoff: <10). Using LIA, one laboratory noted the presence of weak reactivity with AMA-M2, Sm, and Sm/RNP (see Table 1).

IP using ^35^S-methionine-labeled K562 cells confirmed that MND-REF contained antibodies that reacted protein bands corresponding to kelch-like protein 7 (KLHL 7), hexokinase 1 (HK1), and pyruvate dehydrogenase complex (PDC) subunits: E2, E1α, and E1β (Figure 1C, left panel), and this data was further confirmed in IP-MS. There was a major band corresponding to 130 kDa and other unidentified minor bands detected by IP (Figure 1C). However, neither Sp100 nor PML were identified by WB in MOLT4 or HeLa cell lysates (Figure 1D), IP, and IP-MS. The typical process used in generating reference material includes a lyophilization step after dispensing the material into small aliquots for distribution. No differences by HEp-2 IFA, IP (Figure 1C, right panel), LIA, ELISA or ALBIA were observed between MND-REF pre- and post-lyophilization samples. Thus, lyophilization did not appear to show any effect on assay performance.

### GWB-REF validation

**Figure 2:**
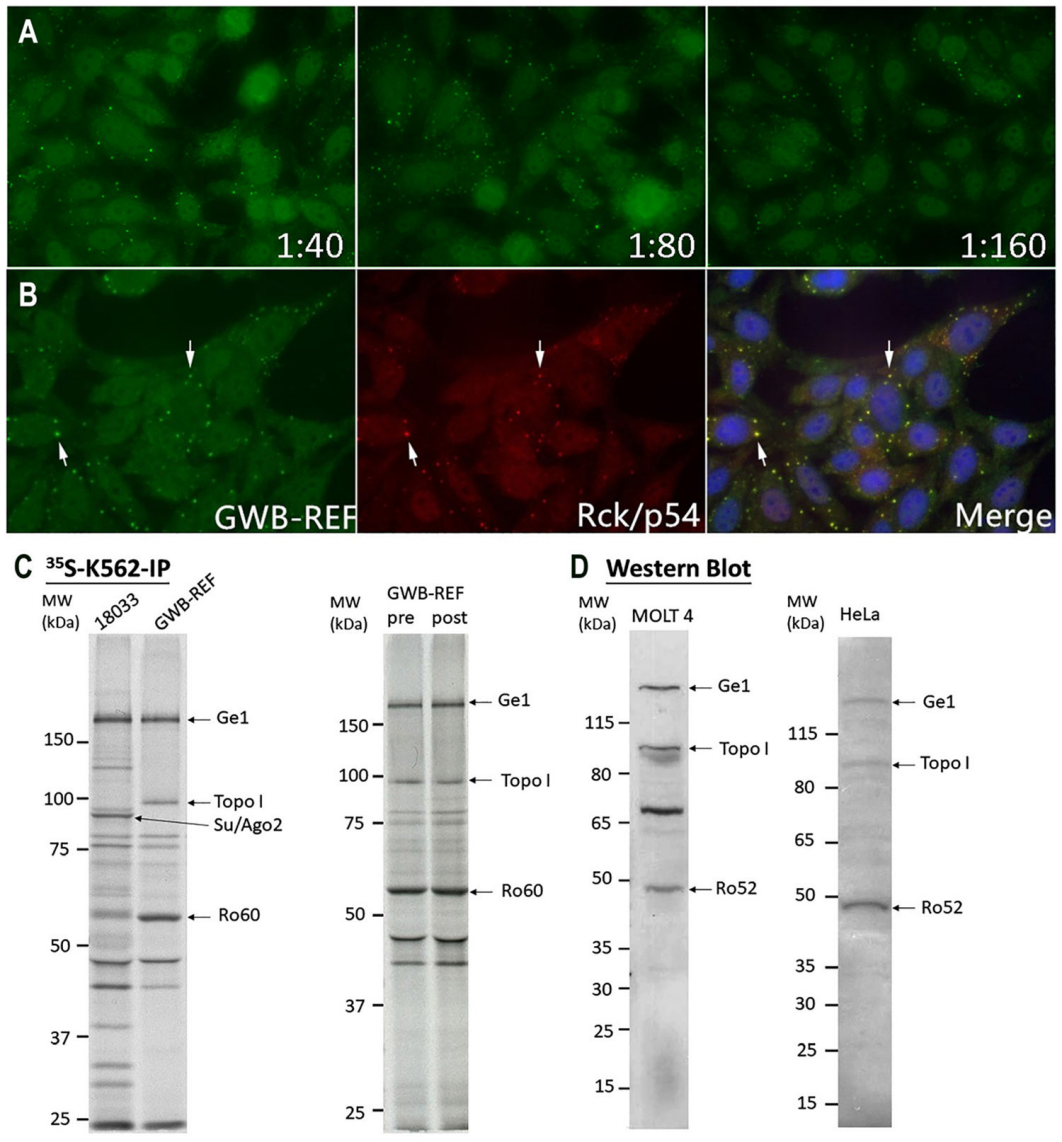
GWB-REF validation by HEp-2 IFA, double staining, ^35^S-K562-IP and western blotting. (A) GWB-REF was serially diluted from 1:40 to 1:1,280 for HEp-2 IFA. Representative images are shown for 1:40, 1:80, and 1:160 dilutions and they all presented discrete cytoplasmic dots corresponding to the ICAP AC-18 pattern (https://www.anapatterns.org/view_pattern.php?pattern=18). At 1:40 dilution, some nuclear staining is also observed. (B) Double staining of cytoplasmic foci (arrows) of GWB–REF (green) and rabbit anti-Rck/p54 (red). (C) Left panel: IP analysis of GWB–REF as described in Figure 1C. Right panel: IP comparison of GWB-REF pre- and post-lyophilization. The proteins immunoprecipitated by GWB-REF were recognized as Ge-1, topoisomerase I and SSA/Ro60 corresponding proteins identified by IP-MS. (D) Western blotting of GWB-REF using MOLT4 (left panel, 1:2,000) or HeLa (right panel, 1:5,000) whole cell lysates. Both of them showed the reactivity to Ge-1, topoisomerase I and Ro52 corresponding to proteins identified by IP-MS.

GWB-REF was first validated by HEp-2 IFA (Figure 2A). The cytoplasmic discrete dots staining in interphase cells with higher numbers in late S/G2 cells is characteristic of the AC-18 pattern, a finding confirmed by all participating laboratories. Serial dilution showed that the antibody titer ranged from 1:160 to ≥1:2,560. In addition, one laboratory also reported DNA topoisomerase I (topo I)-like pattern (AC-29) with titer of 1:160 on Bion, Inova, and homemade HEp-2 slides (Supplementary Table S1). AC-29 is the staining pattern for anti-Topo I as defined by five components [40, 41] and one of which is nuclear fine speckled (AC-4). Many other laboratories observed only the nuclear fine speckled pattern (AC-4) with titer between 1:40 to 1:320 in Bio-Rad, Inova, MBL, and Zeus slides (Supplementary Material, Table S1, Figure S2).

Two color-immunofluorescence using HEp-2 cells confirmed that GWB-REF reacted with GWB co-stained by rabbit anti-Rck/p54 antibodies (Figure 2B). Transfection of plasmids encoding GWB markers GFP-EDC4, GFP-GW182, and GFP-LSm14 into HEp-2 cells and subsequent staining with the reference serum and anti-GFP antibodies further showed that GWB-REF contains antibodies that react with GWB bodies (DBB, data not shown).

In IP, the reactivity of GWB-REF was compared sideby-side with the well-characterized human anti-GWB serum 18033 as a positive control [42-44]. Antibodies in human serum 18033 reacted with multiple GWB-associated proteins including Ge-1 and Ago2 (Figure 2C). GWB-REFimmunoprecipitated Ge-1 was clearly visualized as a ~165 kDa band comigrating as the one immunoprecipitated by serum 18033. Another two bands of approximately 100 and 60 kDa corresponding to topoisomerase I (Scl-70) and SSA/Ro60, respectively, were detected in both pre- and post-lyophilization samples and confirmed in IP-MS analysis. In WB with both MOLT-4 and HeLa whole cell lysates, both bands corresponding to Ge-1 and topoisomerase I were detected. Ro52/TRIM21, but not SSA/Ro60, was detected by WB (Figure 2D).

To check for specificity, GWB-REF reactivity was also analyzed by LIA and ALBIA. Euroline ANA Profile 5, SSc-LIA, Profile Autoimmune Liver Diseases, and Autoimmune Inflammatory Myopathies commercial LIA kits were used. Anti-topoisomerase I, anti-Ro52, and anti-SSA/Ro60 were detected by LIA. Using ALBIA, antibodies in GWB-REF reacted strongly with Ge-1 (MFI: 7358), but reactivity with GW182 or Ago2 were below cut-off (MFI: 53 and 13, respectively). As a quality control, human serum 18033 was tested by ALBIA and was found to contain antibodies that reacted strongly with GW182 (MFI: 9426), Ago2 (MFI: 10458), and Ge-1 (MFI: 5781). One laboratory detected anti-Scl-70 and anti-Ro52 antibodies by ELISA in GWB-REF and the presence of these antibodies was confirmed using double immunodiffusion against calf spleen extract (LECA).

### NuMA-REF validation

**Figure 3:**
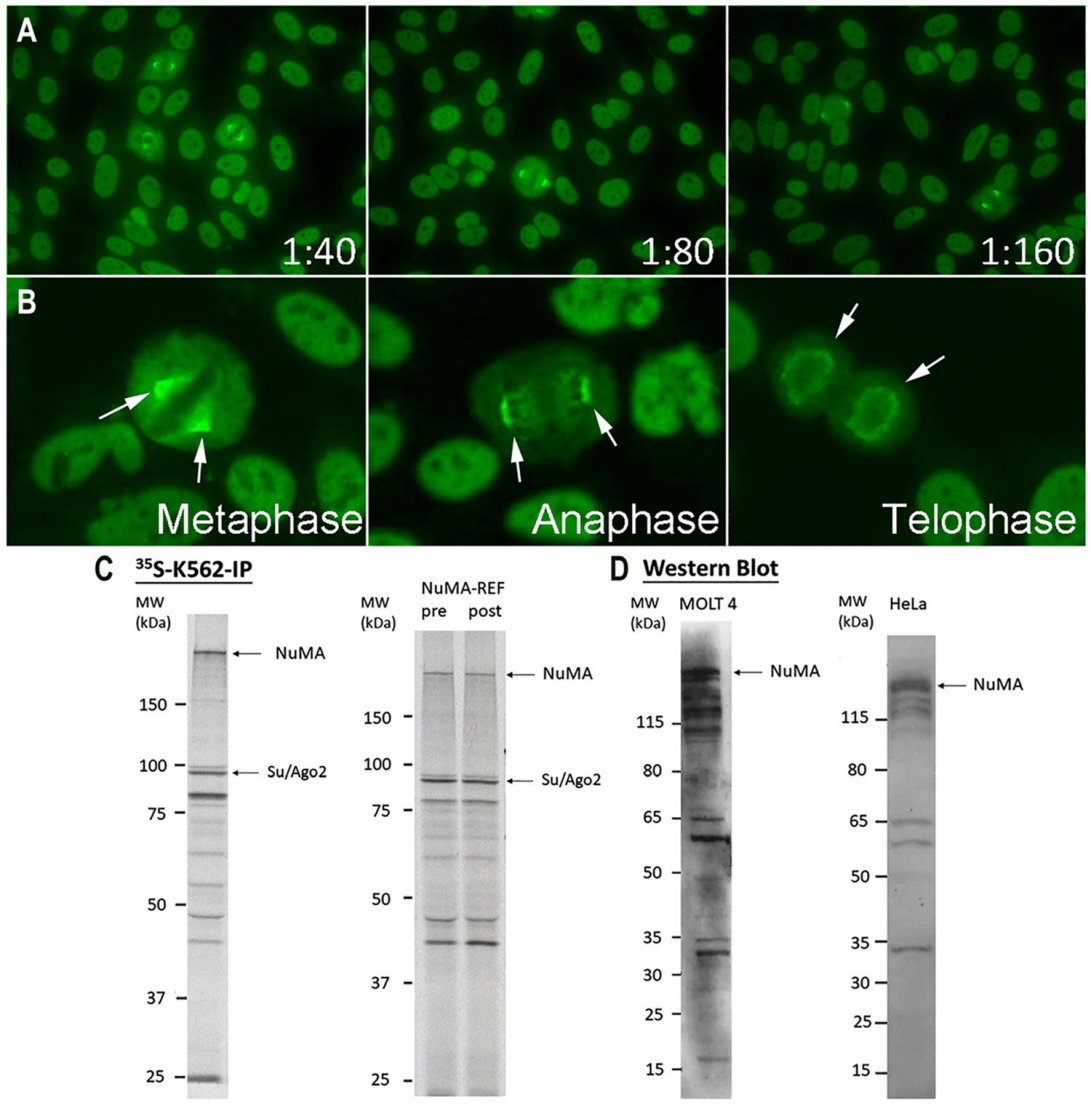
NuMA-REF validation by HEp-2 IFA, ^35^S-K562-IP, and western blotting. (A) Images of NuMA-REF by HEp-2 IFA using 1:40, 1:80, and 1:160 dilutions presenting fine speckled nuclear staining in interphase cells and bright fluorescence of spindle fibers and spindle poles characteristic of the ICAP AC-26 pattern (https://www.anapatterns.org/view_pattern.php?pattern=26). (B) Selected images of metaphase, anaphase, and telophase cells illustrating characteristic NuMA staining. (C) Left panel: IP analysis of NuMA–REF as described in Figure 1C. Right panel: ^35^S-K562-IP of NuMA-REF pre- and post-lyophilization. (D) Left panel: Western blotting of MOLT4 whole cell lysate with NuMA-REF diluted to 1:2,000. Right panel: Western blotting of HeLa whole cell lysate with NuMA-REF diluted to 1:5,000. Both of them showed the reactivity to NuMA.

NuMA-REF was first validated by HEp-2 IFA using Inova slides (Figure 3A). The NuMA-like staining corresponding to the ICAP AC-26 pattern was confirmed by all participating laboratories. Serial dilution showed titers ranging from 1:320 to >1:640. As shown in Figure 3B, in addition to the fine nuclear speckled staining in interphase cells, the characteristic strong staining of mitotic spindles in metaphase cells with weaker staining in anaphase and telophase cells shifting to the periphery of the chromatin plate as described by Andrade et al. [30].

In IP, NuMA-REF reacted with a high molecular weight (>200 kDa) band consistent with the NuMA protein (Figure 3C, left panel), which has a calculated molecular mass of 238 kDa. Both NuMA-REF samples pre- and post-lyophilization were compared by HEp-2 IFA, IP (Figure 3C, right panel), ALBIA, and LIA and no effect of lyophilization on assay performance was observed. In accordance with previous studies [30], a high molecular weight polypeptide was recognized in both MOLT-4 and HeLa cell lysates by the NuMA-REF on WB (Figure 3D). In addition to the strong NuMA band, lower molecular weight bands, presumably corresponding to NuMA degradation products, were also visible [45]. Also, the typical 100 kDa doublet of Su/Ago2 was seen by IP (Figure 3C). In addition to NuMA being identified by IP-MS, Heat shock cognate 71 kDa protein (HSPA8), heat shock protein HSP 90-beta (HSP90AB1), and isoform 2 of heat shock protein HSP 90-alpha (HSP90AA1) were also identified as likely autoantigens but no further validation experiments were performed.

The presence of anti-NuMA antibodies in the reference material was also confirmed by LIA, ALBIA, and ELISA. Antibodies in the serum reacted with NuMA and γ-tubulin by ALBIA and did not react with any of the other antigens present in these assays (Table 1).

## Discussion

The ultimate goal of the Autoantibody Standardization Committee in developing reference materials is to promote standardization of autoantibody testing. The three new reference materials reported here are intended to improve clinical assays, primarily in IFA, to detect these autoantibodies. These three reference materials could be valuable in clinical or research laboratories based on the validation results in our study (Table 2). Note that commercial kits employed in this study were used mainly in individual participating clinical laboratories and do not represent endorsement for their uses.

With MND-REF, Sp100 and PML showed strong positive reaction by LIA method, and Sp100 was also confirmed by ELISA and ALBIA. Many investigators have shown the co-existence of antibodies to Sp100 and PML [8, 16], but the mechanisms leading to the induction of these autoantibodies remain unknown [8, 10]. However, the simultaneous presence of anti-PML and anti-Sp100 antibodies may serve to identify a subgroup of PBC patients who are AMA-negative [9,17]. Additionally, the reference material contained low titer antibodies directed against AMA-M2, anti-Sm, and anti-Sm/RNP as detected by LIA. Antibodies directed against Sm and U1-RNP were not detected by ELISA. The pyruvate dehydrogenase antigen complex (E2, E1α and E1β) recognized by AMA was also detected, while Sm and U1-RNP were negative by WB, IP, and IP-MS. Notably, cytoplasmic reticular/AMA pattern (AC-21) was also reported in three participated laboratories by HEp-2 IFA. As AMA-M2 is the serological hallmark of PBC and is detectable in more than 75% PBC patients, it is common to observe AMA in anti-Sp100 and PML positive serum [37, 46]. However, in ^35^S-K562-IP a strong band at ~130 kDa was visualized which might corresponded to PIC1/SUMO-1-modified PML as reported by Sternsdorf et al. [47], while Sp100 and PML were not detected by WB or IP-MS in both MOLT-4 and HeLa whole cell lysates. This may be due to the characteristic poor solubility of PML and Sp100 proteins. To address this issue, Sternsdorf et al. established a cell line with inducible high levels of PML protein by transfection of HtTA-1 cells with a PML expression vector to perform IP [8]. Given these known limitations, laboratories that wish to confirm the presence of antibodies directed against Sp100 and PML should focus on LIA, ELISA, or ALBIA.

Interestingly, anti-KLHL 7 and anti-HK1 were also demonstrated in MND-REF by IP-MS. KLHL 7 is one of the 42 KLHL family members composed of a BTB/POZ domain, a BACK domain, and 5–6 Kelch motifs [48]. In the KLHL family, autoantibodies to KLHL 7 and 12 have been reported to be associated with autoimmune diseases, including PBC [49-51]. Uchida K et al. identified these two KLHL proteins as novel autoantigens in SjS by phage-display cloning [49]. HK1 is a mitochondrial enzyme that regulates crucial cellular processes [52]. Anti-HK1 and anti-KLHL 12 have been reported as novel biomarkers for PBC patients [49, 51, 53], although their specificity to PBC have not been validated [50].

Cytoplasmic discrete dots/GWB-like pattern (AC-18) are associated with antibodies to GW182, Ge-1, and Su/Ago2. Most research has focused on GW182, even though Ge-1 is the most common autoantigen target in sera demonstrating the GWB/AC-18 HEp-2 IFA pattern [54, 55]. Ge-1 is a 1401-amino-acid protein that is vital to the integrity of GWB. It contains a C-terminal domain which has a repeating ψ(X_2–3_) motif directing the protein GWB. The GWB-REF recognized predominantly Ge-1 and not GW182. Of note, anti-Ro52 has been reported to co-exist in patients with anti-GWB autoantibodies, and it was also detected in the GWB-REF serum [28, 54]. Since the donor patient had a diagnosis of SSc, it was not surprising that anti-Scl-70/topoI autoantibodies were detected in this reference material.

Although anti-NuMA antibodies are detected with relatively low frequency in routine diagnostic laboratories, the presence of these antibodies is strongly indicative of systemic autoimmune diseases, including SLE and SjS. The ability to detect these antibodies may be especially useful when patients do not have the more common disease-associated antibodies, such as those directed against SSA/SSB or dsDNA [31, 33]. In addition, anti-NuMA antibodies may also be present in patients with non-autoimmune conditions such as osteoarthritis, cancer, and certain infections [56, 57]. Because there are no commercially available analyte-specific kits for detection of anti-NuMA antibodies and in some cases anti-NuMA sera may be negative by IP [30], HEp-2 IFA is currently the main methodology used by clinical laboratories to detect and report the NuMA-like AC-26 pattern. ICAP assigned AC-26 as an expert-level reporting, which means this “composite” pattern to some extent could not be easily recognized or is underreported or not reported at all by some inexperienced laboratories. The availability of NuMA-REF should be useful for expertise building in recognition of AC-26 pattern. Moreover, the presence of anti-NuMA antibodies in NuMA-REF was validated by IP-MS, WB, ALBIA, and ^35^S-K562-IP, which indicated that it also could be a reference material for research purposes.

In summary, this paper reports on the immunoreactivity of three novel reference materials for autoantibodies against MND, GWB, and NuMA respectively, using a variety of immunological methodologic platforms. Aliquots of these reference materials will be available at PSG to all qualified academic and commercial clinical laboratories for the purpose of improving standardization and quality control of autoantibody testing.

## Supplementary Material

Suppl materials

## Figures and Tables

**Table 1:** Summary of LIA, ALBIA, and ELISA results for MND, GWB, and NuMA reference materials.

Reference materials	Assay	Positive antigens	Negative/excluded antigens
MND-REF AC-6	LIA	Sp100, PML	nRNP/Sm^a^, Sm^a^, RNP-70, RNP-A, RNP-C, SSA/Ro60, Ro52, SSB, Scl-70, PM/Scl, Jo-1, CENP-B, PCNA, dsDNA, nucleosomes, histones, Rib-P, RP155, RP11, fibrillarin, Th/To, NOR90, PDGFR, CENP-A, Ku, PM/Scl75, PM/Scl100, AMA-M2^a^, M2-3E (BPO), gp210, LKM-1, LC-1, SLA/LP, Mi-2α, Mi-2β, TIF1 γ, MDA-5, NXP2, SAE1, SRP, PL-7, PL-12, EJ, OJ
	ALBIA	Sp100, Ago2	p80-coilin, SMN1, Gemin3, RUVBL1, RUVBL2, GW182, Ge-1, EEA1, HsEg5, γ-Tubulin, PCNT, CENPF1, CENPF4, NuMA, Enolase1, PLK4, Cep110, Ninein, AMA-Mit3, LKM, SLA, LC1, HK, KL, gp210, VCP
	ELISA	Sp100	dsDNA, Sm, U1-RNP, SSA/Ro60, SSB, Jo-1, Scl-70
GWB-REF AC-18	LIA	Scl-70, SSA/Ro60^b^, Ro52^c^	nRNP/Sm, Sm, RNP-70, RNP-A, RNP-C, SSB, PM/Scl, Jo-1, CENP-B, PCNA, dsDNA, nucleosomes, histones, Rib-P, RP155, RP11, fibrillarin, Th/To, NOR90, PDGFR, CENP-A, Ku, PM/Scl75, PM/Scl100, AMA-M2, M2-3E (BPO), Sp100, PML, gp210, LKM-1, LC-1, SLA/LP, Mi-2α, Mi-2β, TIF1γ, MDA-5, NXP2, SAE1, SRP, PL-7, PL-12, EJ, OJ
	ALBIA	Ge-1, Cep110	p80-coilin, SMN1, Gemin3, RUVBL1, RUVBL2, GW182, Ago2, EEA1, HsEg5, γ-Tubulin, PCNT, CENPF1, CENPF4, NuMA, Enolase1, PLK4, Ninein, AMA-Mit3, LKM, SLA, LC1, HK, KL, Sp100, gp210, VCP
	ELISA	Scl-70	dsDNA, Sm, U1-RNP, SSA/Ro60, SSB, Jo-1, Sp100
NuMA-REF AC-26	LIA	ND	nRNP/Sm, Sm, RNP-70, RNP-A, RNP-C, SSA/Ro60, Ro52, SSB, Scl-70, PM/Scl, Jo-1, CENP-B, PCNA, dsDNA, nucleosomes, histones, Rib-P, AMA-M2
	ALBIA	NuMA, γ-Tubulin	p80-coilin, SMN1, Gemin3, RUVBL1, RUVBL2, GW182, Ge-1, Ago2, EEA1, HsEg5, PCNT, CENPF1, CENPF4, Enolase1, PLK4, Cep110, Ninein, AMA-Mit3, LKM, SLA, LC1, HK, KL, Sp100, GP210, VCP
	ELISA	ND	dsDNA, Sm, U1-RNP, SSA/Ro60, SSB, Jo-1, Scl-70, Sp100

ND, none detected.

a reported only as a weak positive in one lab and was not positive in IP.

b SSA/Ro60 confirmed in IP (Figure 2).

c Ro52 confirmed in WB (Figure 2).

**Table 2:** Applications of MND-REF, GWB-REF, and NuMA-REF in clinical and research laboratories.

Methods	MND-REF	GWB-REF	NuMA-REF
Clinical lab			
HEp-2 IFA	AC-6	AC-18	AC-26
LIA	Sp100, PML	Scl-70/topo I, SSA/Ro60, Ro52	NR
ELISA	Sp100	Scl-70/topo I	NR
Research lab			
^33^S-K562-IP	AMA	Ge-1, Scl-70/topo I, SSA/Ro60	NuMA, Su/Ago2
WB	NR	Ge-1, Scl-70/topo I	NuMA
IP-MS	NR	Ge-1, Scl-70/topo I	NuMA
ALBIA	Sp100	Ge-1	NuMA, γ-Tubulin

NR, no recommendation.
