# Supplementary material for: Establishment of international autoantibody reference standards for the detection of autoantibodies directed against PML bodies, GW bodies, and NuMA protein
Source: Clin Chem Lab Med. Author manuscript; Available in PMC 2021 Feb 3. (PMC7855248; doi:10.1515/cclm-2020-0981)
Supplement: Suppl materials [file NIHMS1644893-supplement-Suppl_materials.pdf]

Supplemental Materials. Zheng et al.

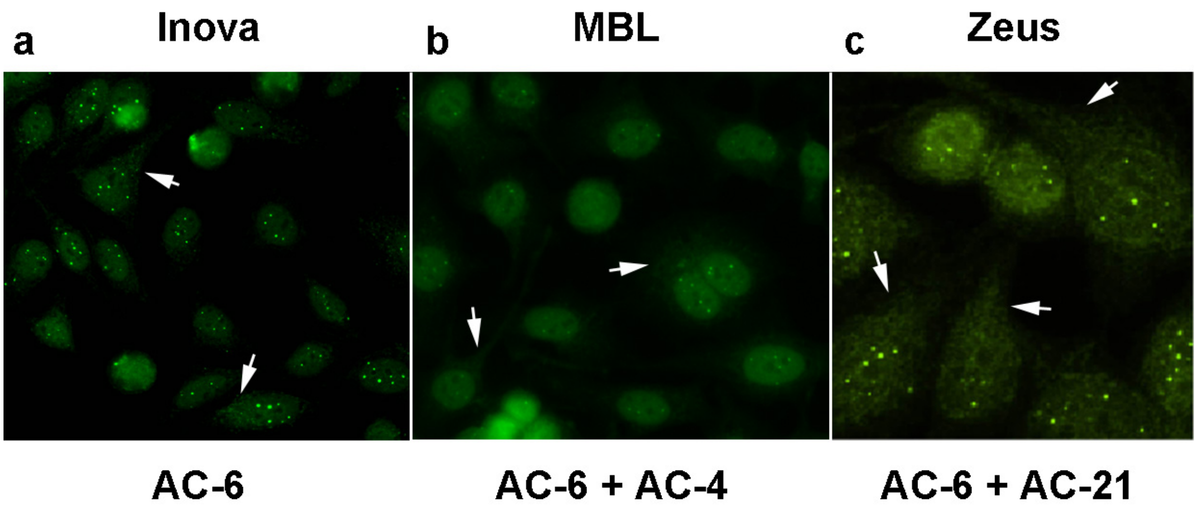

Figure S1. Representative IFA images reported for MND-REF from different laboratories as summarized in Table S1. All laboratories reported a consensus on multiple nuclear dots (AC-6) typical as shown in all panels. Some laboratories reported AC-6 + AC-4 (nuclear fine speckled) as illustrated in panel **b** while other laboratories reported AC-6 + AC-21 (cytoplasmic reticular/AMA) as shown in panel **c**. On careful examination, AC-4 can be seen in all panels to varying degree and hints of AC-21 (arrows) seen in some cells in both panel **a** and **b** as well.

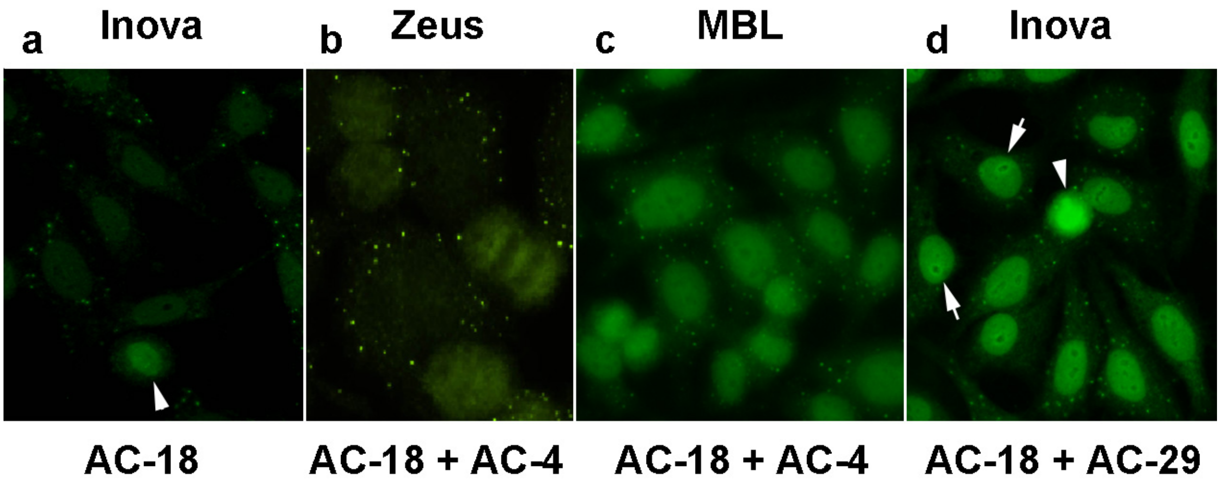

Figure S2. Representative IFA images reported for GWB-REF from different laboratories as summarized in Table S1. All laboratories reported a consensus on cytoplasmic discrete dots (AC-18) typical as shown in these panels. Some laboratories reported AC-18 + AC-4 (nuclear fine speckled) as illustrated in panel **b** and **c** while other reported AC-18 + AC-29 (DNA topoisomerase I-like) as shown in panel **d**. Features for AC-29 include AC-4, condensed chromatin staining in mitotic cells (arrowhead), strong staining of nucleolar organizing region (not shown), weak cytoplasmic staining, and variable nucleolar staining (perinucleolar, arrows).

Table S1. Summary of HEp-2 IFA results in participating laboratories using different HEp-2 slides.

**Laboratory**	**HEp-2 cell substrate**	**Secondary antibodies**	**MND-REF**	**GWB-REF**	**NuMA-REF**
Andrade	Bion	FITC labeled goat anti-human IgG (H+L) with Evans Blue counterstain (CCP-9920, MBL-Bion)	AC-6 (≥1/640); AC-4 (1/640); AC-21 (1/320)	AC-18 (1/640); AC-29 (1/160)	AC-26 (≥ 1/640)
	Inova	FITC IgG conjugate with DAPI premixed solution (508102, Inova)	AC-6 (≥1/640); AC-20 (1/320)	AC-18 (1/640); AC-29 (1/160)	AC-26 (≥ 1/640)
	In house	Same conjugate as in Bion	AC-6 (≥1/640); AC-4 (1/320)	AC-18 (1/640); AC-29 (1/160)	AC-26 (≥1/640)
Bloch	Zeus	Alexa Fluor 488 AffiniPure donkey anti-human IgG (H+L, 709-545-149, Jackson ImmunoResearch)	AC-6 (≥1/2560); AC-21	AC-18 (≥1/2560); AC-4	AC-26 (≥1/2560)
Chan	Inova	Alexa Fluor 488-conjugated goat anti-human IgG (H+L, A11013, Thermo Fisher Scientific)	AC-6 (≥1/1280); AC-4 (1/320)	AC-18 (1/640); AC-4 (1/160)	AC-26 (1/640)
Fritzler	Inova	FITC IgG conjugate with DAPI premixed solution (508102, Inova)	AC-6 (>1/640)	AC-18 (>1/640)	AC-26 (>1/640)
Satoh	MBL	Alexa Fluor 488-conjugated AffiniPure goat anti-human IgG, (gamma-chain specific, 109-546-170, Jackson ImmunoResearch)	AC-6 (≥1/320); AC-4 (≥1/320)	AC-18 (≥1/320); AC-4 (≥1/320)	AC-26 (≥1/320)
Wener	Bio-Rad	FITC tagged sheep anti-human immunoglobulin antibodies (Bio-Rad)	AC-6 (1/320); AC-21 (1/320)	AC-18 (1/160); AC-4 (1/40)	AC-26 (1/320)
